# A Study on the Compressive Behavior of Additively Manufactured AlSi10Mg Lattice Structures

**DOI:** 10.3390/ma17215188

**Published:** 2024-10-24

**Authors:** David Liović, Sanjin Kršćanski, Marina Franulović, Dražan Kozak, Goran Turkalj, Emanuele Vaglio, Marco Sortino, Giovanni Totis, Federico Scalzo, Nenad Gubeljak

**Affiliations:** 1 University of Rijeka, Faculty of Engineering, Vukovarska 58, 51000 Rijeka, Croatia; sanjin.krscanski@uniri.hr (S.K.); marina.franulovic@uniri.hr (M.F.); goran.turkalj@uniri.hr (G.T.); 2 Mechanical Engineering Faculty in Slavonski Brod, University of Slavonski Brod, Trg I. B. Mažuranić 2, 35000 Slavonski Brod, Croatia; dkozak@unisb.hr; 3 Polytechnic Department of Engineering and Architecture, University of Udine, Via delle Scienze 206, 33100 Udine, Italy; emanuele.vaglio@uniud.it (E.V.); sortino@uniud.it (M.S.); giovanni.totis@uniud.it (G.T.); federico.scalzo@uniud.it (F.S.); 4 Faculty of Mechanical Engineering, University of Maribor, Smetanova 17, 2000 Maribor, Slovenia; nenad.gubeljak@um.si

**Keywords:** lattice structure, BCC, compressive behavior, additive manufacturing, AlSi10Mg

## Abstract

The mechanical behavior of the metallic components fabricated by additive manufacturing (AM) technologies can be influenced by adjustments in their microstructure or by using specially engineered geometries. Manipulating the topological features of the component, such as incorporating unit cells, enables the production of lighter metamaterials, such as lattice structures. This study investigates the mechanical behavior of lattice structures created from AlSi10Mg, which were produced using the laser beam powder bed fusion (LB-PBF) process. Specifically, their behavior under pure compressive loading has been numerically and experimentally investigated using ten different configurations. Experimental methods and finite element analysis (FEA) were used to investigate the behavior of body-centered cubic (BCC) lattice structures, specifically examining the effects of tapering the struts by varying their diameters at the endpoints ($d_{\mathrm{end}}$) and midpoints ($d_{\mathrm{mid}}$), as well as altering the height of the joint nodes (*h*). The unit cells were designed with varying parameters in such a way that $d_{\mathrm{end}}$ is changed at three levels, while $d_{\mathrm{mid}}$ and *h* are changed at two levels. Significant differences in Young’s modulus, yield strength, and ultimate compressive strength between the various specimen configurations were observed both experimentally and numerically. The FEA underestimated the Young’s modulus corresponding to the configurations with thinner struts in comparison to the higher values found experimentally. Conversely, the FEA overestimated the Young’s modulus of those configurations with larger strut diameters with respect to the experimentally determined values. Additionally, the proposed FE method consistently underestimated the yield strength relative to the experimental values, with notable discrepancies in specific configurations.

## 1. Introduction

Additive manufacturing (AM) has opened up numerous possibilities in the production of lattice structures from metallic materials, which are characterized by pronounced topological complexity [1,2,3]. The lattice structures consist of unit cells that can be body-centered (BCC), face-centered (FCC), cubic (SC), tetrahedral, pyramidal, octahedral, Kagome, and others [4]. They have great potential for use in various fields, such as the automotive and aerospace industries, as well as in biomedicine [5]. Lattice structures are characterized by a high strength-to-weight ratio, low mass due to their partially void structure, and the ability to tailor the mechanical response for specific purposes [5]. The mechanical properties of the lattice structures depend mostly on the type and size of the individual characteristics of the unit cells, such as the angle, length, and diameter of the struts and the material from which they are created [6,7,8]. One of the widely used materials for the production of lattice structures is the AlSi10Mg alloy. This alloy can be successfully produced using AM technologies such as laser beam powder bed fusion (LB-PBF) [9], electron beam powder bed fusion (EB-PBF) [10], and laser-engineered net shaping (LENS), where the metal can be supplied in the form of a powder or wire [11].

The mechanical behavior of the metallic components produced using AM technologies can be influenced to the greatest extent by modifying the microstructure or by changing the topological features of the component itself, such as implementing unit cells. Płatek et al. analyzed the influence of the lattice structure density on the damage mechanisms of BCC lattice structures created from 316 L material [12]. They found that lattice structures of higher density are characterized by tension–compression as the dominant damage mechanism under both quasi-static and dynamic loading conditions [12]. Similarly, they found that lattice structures of lower density are characterized by bending as the dominant damage mechanism. Jin et al. analyzed the effect of heat treatment on BCC and FCC lattice structures and found that the BCC lattice structure is more sensitive to heat treatment conditions than the FCC lattice structure [4]. This further opens up the possibility of configuring the mechanical properties of the BCC lattice structure by simultaneously controlling the heat treatment parameters and selecting the geometric features of the unit cells. The mechanical properties of lattice structures can depend on the number of unit cells, so it is necessary to select a sufficient number of unit cells to properly characterize the mechanical behavior of the lattice structure. Smith et al. analyzed the influence of the number of unit cells on mechanical properties and found that most mechanical properties can be simulated using a smaller number of unit cells when the lattice structure is non-stochastic [13]. Yang et al. investigated the influence of the number of unit cells on auxetic lattice structures and found that the elastic modulus value remains constant when the number of cells is equal to or greater than three [14]. Peng et al. found that the number of unit cells in the BCC structure has no significant influence on the material’s elastic behavior, while the results diverge after the yield point [7]. Pelegatti et al. demonstrated that lattice specimens with 6x6 unit cells along the cross section are adequate to characterize the low cycle fatigue mechanical behavior [15]. Zhao et al. investigated the influence of strut tapering on the mechanical properties of BCC latices manufactured from Ti6Al4V alloy powder [16]. They reported that the tapering helps to reduce anisotropy while enhancing the elastic modulus [16]. It is important to point out, however, that the tapering was focused on decreasing the size of the hexagonal base at the midpoint of the strut compared to its size at the node region. Bai et al. advanced the reduction in the stress concentrations at the nodes by implementing graded-strut body-centered cubic (GBCC) structures [6]. Their findings showed that increasing the corner radii of BCC unit cells effectively reduces stress and improves the mechanical properties of the structure [6].

The mechanical behavior of lattice structures under compressive loading has been widely studied by researchers. However, the impact of tapering and joint node height on compressive performance has received little attention. Gaining deeper insights into these factors could offer an advantage in designing advanced lattice structures with customized mechanical properties. For this reason, this work investigated the effect of tapering diameter and joint node height on the Young’s modulus (*E*), yield strength ($R_{p0.2}$), and ultimate compressive strength ($R_{m}$) of AlSi10Mg alloy BCC lattice structures fabricated using LB-PBF. The study involved quasi-static compressive tests on 3D-printed specimens and comparisons with numerical analyses based on the Johnson–Cook damage model.

## 2. Materials and Methods

Considering that this research focuses on lattice structures subjected to quasi-static loading, involving complex contacts and considering material damage, the Explicit solver within the Abaqus 2022 software package was chosen. The Johnson–Cook damage model was utilized for modeling damage, based on which damage initiation was defined. Once the plastic deformation in the finite element exceeds the specified threshold, the finite element is removed from the simulation. The nonlinear analysis conducted using Abaqus also incorporates geometric nonlinearity. This inclusion accounts for the effects of nonlinear material behavior and large deformations, thereby enhancing the accuracy and representativeness of the model. Mass and time scaling methods were employed to reduce the computational time. To mitigate the impact of mass and time scaling on the results, inertial forces were reduced by ensuring that the kinetic energy of the system remained within 5% of its internal energy.

### 2.1. Specimen Design

**Figure 1:**
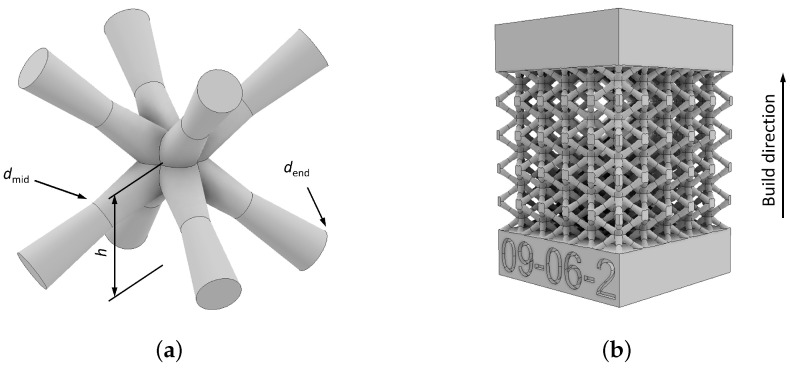
(**a**) Body-centered cubic unit cell with labeled parameters $d_{\mathrm{end}}$, $d_{\mathrm{mid}}$, and *h*; (**b**) lattice structure with $d_{\mathrm{end}}=0.9$ mm, $d_{\mathrm{mid}}=0.6$ mm, and h=2 mm.

The lattice structures investigated in this study consist of a total of 4.6×5×5 body-centered cubic unit cells, as shown in Figure 1a. The single unit cell has a length, width, and height of 4 mm. Consequently, the total height of the lattice portion of the specimen is 18.5 mm, with a length and width of 20 mm. Bulk regions were incorporated at the top and bottom of the lattice structure to ensure a more uniform load distribution during compression testing, as illustrated in Figure 1b.

The incorporation of bulk regions in the lattice structure serves to achieve a more uniform load distribution across each strut. In the absence of these regions, the struts would directly interface with the compression plate of the tensile test machine, leading to potential points of concentrated stress. Given the inherent manufacturing variations and imperfections, the struts are not identical in their dimensions and properties. Consequently, during the application of compressive loads, certain struts may engage earlier and experience higher stress. This non-uniform load distribution can substantially influence the overall mechanical behavior and performance of the lattice configuration. By integrating bulk regions at both the top and bottom of the lattice, a more consistent load distribution is attained. This design modification effectively reduces the risk of localized damage and failure in individual struts that may otherwise be subjected to excessive loading due to manufacturing discrepancies.

When designing lattice structures, the limitations of LB-PBF process, the computational resources required for numerical analysis, and the constraints of the experimental setup were considered. After a preliminary FEA on various configurations and dimensions of lattice structures, a configuration with a lattice of 4.6×5×5 unit cells, each measuring 4×4×4 mm, was selected. This approach provided the necessary basis for a reliable and realistic comparison between the simulation and experimental results. Of course, it is possible to produce lattice structures with a high number of unit cells. However, in that case, numerical simulations become extremely computationally costly. To address this limitation, the total number of unit cells in the FE model can be reduced compared to the actual number in the manufactured specimen [17]. However, this raises an additional concern about the representativeness of the selected number of unit cells, making it necessary to conduct a convergence analysis to evaluate how the number of unit cells influences the mechanical response [17]. To avoid additional influence induced by the differences between the real and simulated structures, this work selected an identical number of unit cells for both the actual lattice structures and the model used for FEA. Accordingly, other geometric features of the lattice structure were defined to ensure its manufacturability. Therefore, the BCC unit cells examined in this study are defined by the following characteristics: the strut end diameter ($d_{\mathrm{end}}$) varies between 0.6 mm, 0.9 mm, and 1.5 mm, while the strut midpoint diameter ($d_{\mathrm{mid}}$) is either 0.6 mm or 0.9 mm. Additionally, the height of the joint nodes (*h*) can be either 2 mm or 3 mm. The $d_{\mathrm{end}}$ levels of 0.6 mm, 0.9 mm, and 1.5 mm and $d_{\mathrm{mid}}$ levels of 0.6 mm or 0.9 mm were selected due to the limitations regarding the available space defined by unit cell size and LB-PBF technology to manufacture some configurations of the BCC unit cells. In the case of asymmetric unit cells when h=3 mm, the impact of unavoidable dross formations on the deviation of the actual strut size from the intended design becomes more significant. This deviation results in pronounced surface defects, irregularities, and increased roughness, which negatively affect the mechanical performance of the lattice structure. Additionally, dross formation makes it challenging to determine the actual strut diameters and geometry after manufacturing as those struts then have highly irregular shapes. The minimum strut diameter was set at 0.6 mm since smaller diameters are challenging to manufacture, being more fragile and prone to defects.

A total of 4.6 unit cells in height was selected to mitigate failure at contact regions between the solid and lattice. This led to shorter struts of boundary unit cells near solid parts, increasing their stiffness and preventing failure at contact regions. A total of 5×5 repeating unit cells in a row (width × length) was selected to reduce the edge effects and enable reliable representation of the whole lattice structure. Edge effects are a well-documented phenomenon [18] that can be mitigated by designing lattices with an appropriate number of cells in each direction [15]. These effects can influence the overall stiffness and strength of the structure [19]. In lattice structures, edge effects refer to variations in mechanical behavior, such as stress distribution and deformation, that occur at the boundaries compared to the interior. In mechanical applications, they can significantly impact performance, especially near the edges, where stress concentrations or irregularities arise due to the absence of neighboring cells. Furthermore, specimen ID embossed on specimens and used within this work takes the following form: $d_{\mathrm{end}}-d_{\mathrm{mid}}-h$. Each different unit cell configuration is summarized in Table 1.

### 2.2. Laser Beam Powder Bed Fusion Process

**Figure 2:**
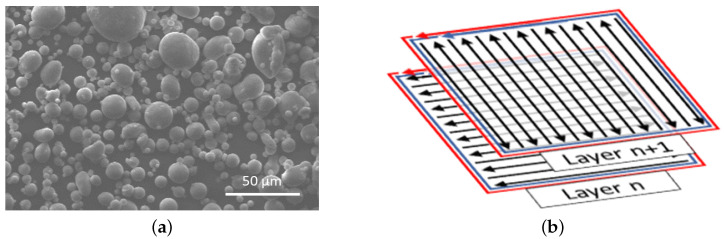
(**a**) SEM view of the AlSi10Mg powder and (**b**) scanning strategy: contour I (blue), contour II (red), and surface (black).

The specimens were fabricated from AlSi10Mg powder using a Concept Laser M2 Cusing apparatus (Concept Laser GmbH, Lichtenfels, Germany) featuring a single-mode continuous-wave ytterbium-doped fiber laser with an emission wavelength (λ) of 1070 nm. The utilized AlSi10Mg powder consisted of spherical particles as shown in Figure 2a, with the chemical composition detailed in Table 2. The 10th, 90th percentile, and median of the powder particle size distribution were 23.2 µm, 48.7 µm, and 27.48 µm, respectively.

The process was executed in an inert argon atmosphere, maintaining the O^2^ content below 0.2%. To maximize the quality of the parts, each layer was divided into three elements, illustrated in Figure 2b: the surface area, one inner contour (contour I), and one outer contour (contour II). The surface was scanned using a bidirectional and alternated pattern, ensuring high and uniform structural performance. Each contour was obtained with a single scan pass that maximized the surface quality. Detailed parameters for each processed element are in Table 3.

The specimens were fabricated in a single batch following the orientation depicted in Figure 1b. The orientation of the reticular structures was optimized to minimize inherent issues resulting from the manufacturing process and obtain the highest-quality parts. Furthermore, the samples were rotated by 45° relative to the recoating direction to facilitate the creation of smooth and seamless powder layers.

**Figure 3:**
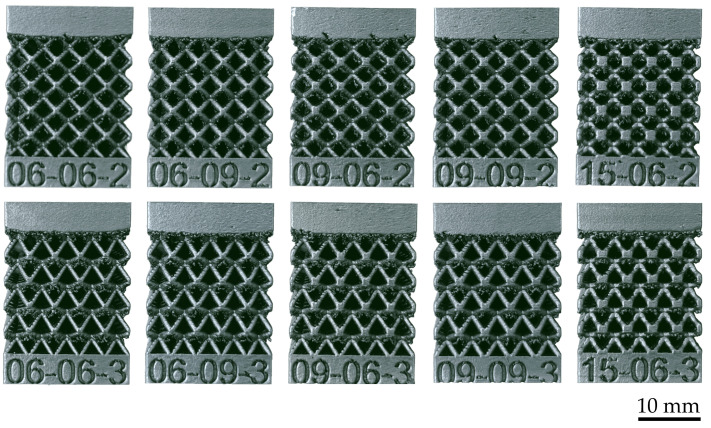
Produced lattice structures.

Following the LB-PBF process, the specimens were annealed in an inert argon atmosphere to reduce residual stresses. Precisely, they were heated to 240 °C in one hour, held 6 h at this temperature, and gradually cooled in the oven until the temperature of 100 °C was reached. The specimens were detached from the build platform using a band saw machine and milled on the upper and lower bulk surfaces to achieve the prescribed flatness tolerances necessary for the compression tests. Lattice structures after manufacturing, heat treatment, and final machining are shown in Figure 3.

## 3. Experimental Tests

**Figure 4:**
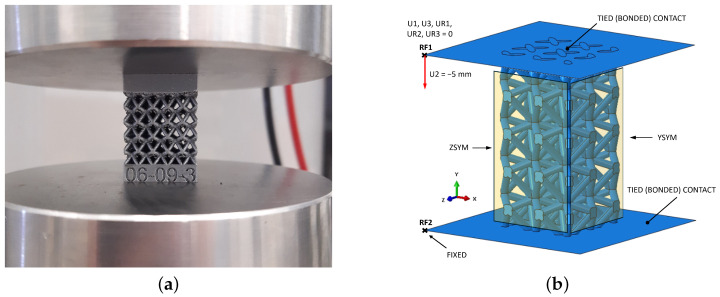
(**a**) Compression test setup and (**b**) FE model with analytically rigid plates, reference points, boundary conditions, and constraints.

Compression tests were conducted using StepLAB electromechanical actuator (STEP Engineering S.r.l., Resana, Italy) equipped with a force sensor of 25 kN capacity and two pressure plates, as shown in Figure 4a. The displacement speed was set to 0.01 mm/s, and strain values were recorded by the absolute encoder. The nominal stress is determined by dividing the load by the nominal cross-sectional area of the BCC lattice specimen considered as bulk material (20×20 mm^2^). Young’s modulus is determined using stress–strain data within the elastic region defined by adjustable upper and fixed lower limits. More specifically, the upper limit can take values from 90% $R_{p0.2}$ to 50% $R_{p0.2}$, while lower limit is kept constant at 15% $R_{p0.2}$. In every iteration, the range is reduced by lowering the upper limit until the coefficient of determination is $R^{2}$ < 0.998. The decision to include an adjustable upper limit was driven by the distinct and significant nonlinear behavior observed in the upper portion of the stress–strain curve, just below the $R_{p0.2}$ values, which varies between different lattice structure configurations.

As can be seen in Table 4, the lowest Young’s modulus, yield strength, and ultimate compressive strength values were found when the unit cells with the smallest strut diameters were used (06-06-2 and 06-06-3).

**Figure 5:**
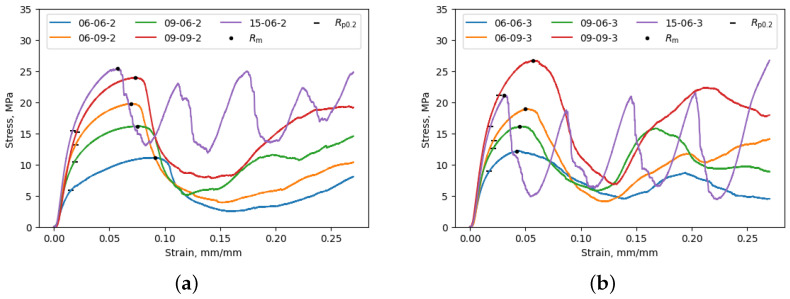
Experimental results for (**a**) specimen configurations with 2 mm node height and (**b**) specimen configurations with 3 mm node height.

Configurations 15-06-2 and 15-06-3 shown in Figure 5 exhibit distinctive cyclic crushing behavior characterized by periodic stress fluctuations. These fluctuations occur due to layer-by-layer collapse and the formation of new contacts between adjacent layers. When comparing configurations 06-09-2 and 09-06-2 in Figure 5a, it becomes evident that the configuration with a larger diameter at the strut midpoint (i.e., 06-09-2) exhibits higher values of $R_{m}$ and $R_{p0.2}$. This trend is also observed in configurations 06-09-3 and 09-06-3, as shown in Figure 5b. As expected, the increase in diameter at the strut midpoint enhances the mechanical performance of the unit cell, particularly in terms of compressive strength.

The specimens with a joint node height *h* equal to 2 mm exhibit higher strains compared to those with *h* equal to 3 mm given the same $R_{m}$. This result can be explained by the different topology of the unit cell. In the case of the cell with *h* equal to 3 mm, the struts’ arrangement promotes the creation of a stiffer cell, which deforms less under the same $R_{m}$. This result confirms that mechanical properties can be customized to meet specific requirements by adjusting the geometric features of the lattice’s unit cell.

## 4. Numerical Analysis

Due to the staircase effect specific to the LB-PBF manufacturing process, the actual diameters of the struts exceed those defined in CAD models. As a result, the actual mean values of strut diameters were higher than those defined prior to manufacturing. Therefore, the models used in FEA have been corrected using actual mean values of strut diameters reported in Table 5. The actual strut diameters were measured 12 times before the compression test using a caliper with a resolution of 0.01 mm, repeatability of 0.01 mm, and a maximum error of 0.02 mm. It is worth noting that the values reported in Table 5 are in good accordance with measurements performed in [20]. While mean strut diameter adjustments were incorporated into the models used in FE simulations, as reported in Table 5, this approach does not consider variations in a cross-sectional area across different regions of the strut, nor the irregularities in shape that may arise due to the LB-PBF process. Additionally, defects in microstructure, such as voids and inclusions typical of the LB-PBF process, were not considered while simulating the compressive behavior of the lattice structures using the finite element method.

To simulate the behavior of the lattice structure under compressive loading, upper and lower plates were added to transmit the load to the observed lattice structure, as can be seen in Figure 4b. For this purpose, a displacement of the upper plate was set to −5 mm, while the remaining 5 degrees of freedom were constrained. The displacement of the upper plate was defined using a smooth step amplitude to reduce the influence of inertial forces at the beginning and end of the simulation. The simulation time period was set to 1 s and the mass scale to 1000. The lower plate was fully fixed at a single point. Reference points were placed on the upper and lower plates, through which the reaction force value for the given displacement is recorded. Engineering stress and engineering strain were calculated in the post-processing phase based on reaction force and displacement data. The nominal area of the lattice specimen (400 mm^2^) was used to calculate engineering stress. Furthermore, a tied (bonded) contact was set between the lattice structure and both plates.

To describe the elastic behavior of the lattice structure subjected to compressive load, Young’s modulus of the parent AlSi10Mg alloy was set to 40 GPa, corresponding to strut thickness of 1 mm, as reported in [21]. Poisson’s ratio was set to 0.3, as reported in [22].

To describe plastic behavior during crushing, the plastic part of the stress–strain curve derived from specimens with strut thickness of 1 mm was used, as reported in [21]. When comparing experimentally observed and simulated crushing behaviors, a greater agreement was achieved when employing mechanical properties derived from specimens with a 1 mm strut thickness as opposed to standardized specimens. More specifically, an intrinsic relationship exists between the mechanical properties of additively manufactured metallic materials and the material thickness, as stated in [21].

To assess damage in lattice structures, the Johnson–Cook damage model was utilized, as described in [23]. In the simulation, the plastic strain at failure $\left({\bar{\varepsilon }}_{f}^{\mathrm{pl}}\right)$ defined with the Johnson–Cook model serves as the threshold value, beyond which damage evolution begins through the degradation of element stiffness:

(1) $${\bar{\varepsilon }}_{f}^{\mathrm{pl}}=\left[d_{1}+d_{2}\exp\left(-d_{3}\eta \right)\right]\left[1+d_{4}\ln\left(\frac{{\dot{\bar{\varepsilon }}}^{\mathrm{pl}}}{{\dot{\varepsilon }}_{0}}\right)\right]\left(1+d_{5}\hat{\theta }\right).$$

where $d_{1}$, $d_{2}$, $d_{3}$, $d_{4}$, and $d_{5}$ are the five material constants in the Johnson–Cook damage model, η is stress triaxiality, $\dot{\varepsilon_{0}}$ is the reference strain rate, ${\dot{\bar{\varepsilon }}}^{\mathrm{pl}}$ is the equivalent plastic strain rate, and $\hat{\theta }$ is the nondimensional temperature. The material constants $d_{1}$, $d_{2}$, and $d_{3}$ in the Johnson–Cook model include the effects of varying stress triaxiality conditions on the plastic strain at failure. This approach considers the different stress states that occur when compressive loads are applied to lattice specimens. The constant $d_{4}$ captures the influence of strain rates on the plastic strain at failure, while $d_{5}$ includes the impact of temperature on the same parameter. In this research, the values of these material constants were selected based on the findings of [24], with $d_{1}$, $d_{2}$, $d_{3}$, $d_{4}$, and $d_{5}$ set to 0, 0.873, −0.449, 0.00147, and 0.8, respectively. Once the equivalent plastic strain in an element surpasses this failure threshold, as defined by Equation (1), the element is immediately removed from the mesh. This instantaneous elimination is achieved by setting the displacement at failure to zero within the damage evolution law, ensuring that elements are removed as soon as the plastic strain at failure is reached.

Mathematically, this is defined as follows. Damage initiation occurs when the accumulated plastic strain reaches a critical value, which usually corresponds to the value of plastic strain when ultimate strength is reached. Therefore, the damage is initiated when the state variable $\omega_{D}$ reaches value of 1:

(2) $$\omega_{D}=\sum \left(\frac{d{\bar{\varepsilon }}^{\mathrm{pl}}}{{\bar{\varepsilon }}_{f}^{\mathrm{pl}}}\right)=1,$$

where $d{\bar{\varepsilon }}^{\mathrm{pl}}$ is the increment of equivalent plastic strain and ${\bar{\varepsilon }}_{f}^{\mathrm{pl}}$ represents the value of plastic strain at failure determined by Equation (1), with summation across all increments. Once the damage is initiated, the damage evolution stage starts, and the overall damage variable *D* is introduced. In this stage, the elastoplastic material’s stress-carrying capability and elasticity are reduced as damage evolves. Therefore, the stress tensor is determined using the scalar damage equation as follows:

(3) $$\sigma =\left(1-D\right)\bar{\sigma },$$

where $\bar{\sigma }$ is the effective (undamaged) stress tensor and $D\in \left[0,1\right]$. When the damage is initiated, usually when the ultimate strength is reached, D=0. As the damage evolves, the *D* increases until it reaches the value of 1, indicating material failure. In this research, the displacement at failure in the damage evolution model was set to 0, indicating that no specific damage evolution law is defined. Consequently, once $\omega_{D}$ reaches a value of 1, the damage variable *D* also immediately reaches 1.

**Figure 6:**
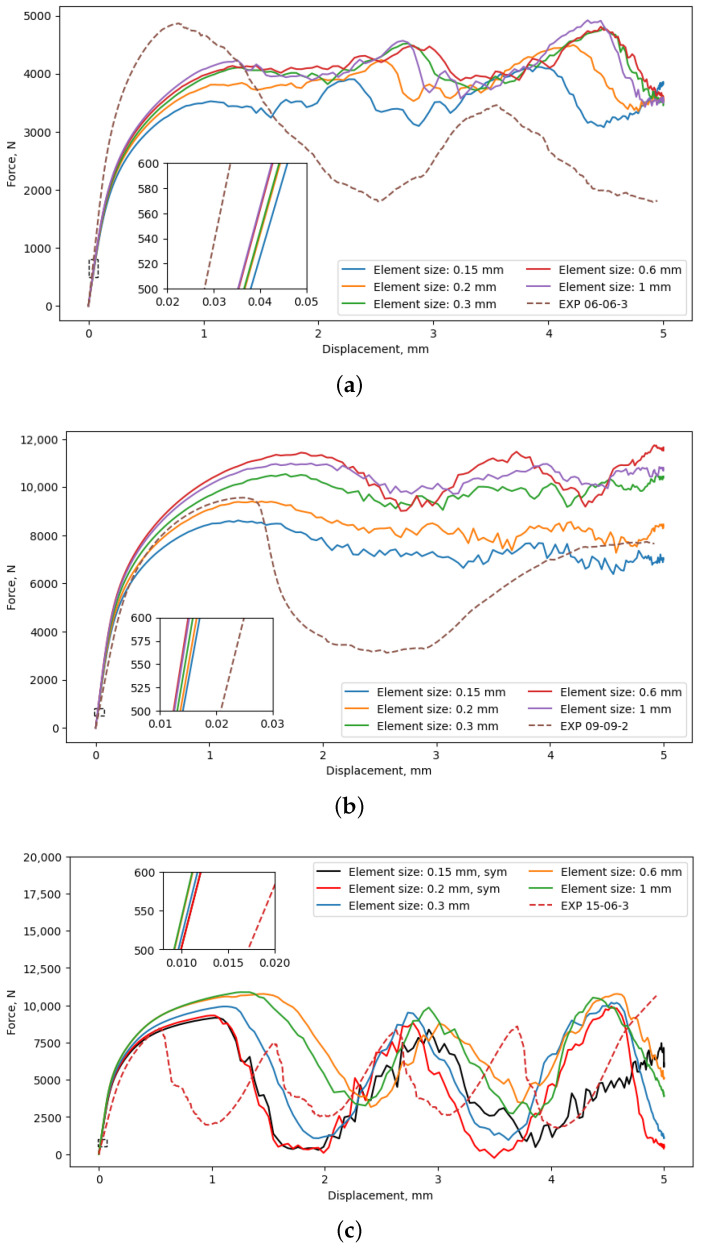
Comparison of load–displacement curves for lattice configurations with different element sizes: (**a**) 06-06-3, (**b**) 09-09-2, and (**c**) 15-06-3.

The convergence analysis was performed to identify appropriate element size and balance accuracy and computational costs. For this purpose, five different element sizes are considered (0.15 mm, 0.2 mm, 0.3 mm, 0.6 mm, and 1 mm), as shown in Figure 6, while resulting mechanical properties are reported in Table 6. It can be seen that element size influences the initial part of the load–displacement curve. However, the deviations in mechanical response within this region are less pronounced than those that occur after the ultimate compressive strength is reached.

**Figure 7:**
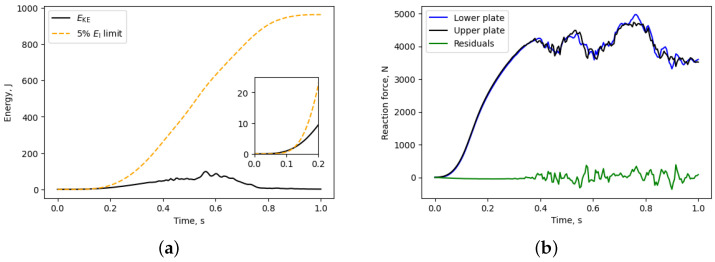
(**a**) Energy and (**b**) reaction force of 06-06-3 specimen in FE simulation.

The ratio between kinetic energy ($E_{\mathrm{KE}}$) and internal energy ($E_{I}$) within the system was monitored in every simulation since time and mass scale procedures were used to reduce computational time. In Figure 7a, it is visible that the achieved value of $E_{\mathrm{KE}}$ is below 5% of the $E_{I}$ value, thereby reducing the impact of inertial forces. Additional control was asserted by comparing the reaction forces on the upper and lower plates, as shown in Figure 7b. It is evident that there is a minimal disparity in reaction force between the two plates, particularly during the early stages of the FE simulation. Nevertheless, around 0.38 s later, when damage arises at specific struts, a disturbance in reaction force becomes apparent. However, this disturbance does not influence calculated values of Young’s modulus, yield strength, and compressive strength as these material properties were derived from the initial part of the stress–strain curve, where damage has not yet occurred.

**Figure 8:**
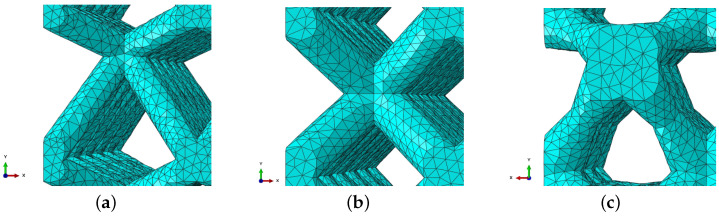
Mesh details for one unit cell of the following lattice configurations: (**a**) 06-06-3, (**b**) 09-09-2, and (**c**) 15-06-3.

Mechanical properties reported in Table 6 were determined from the load–displacement curves by transforming them into stress–strain curves. The differences in Young’s modulus and yield strength between models meshed using an element size of 0.15 mm when compared to an element size of 0.3 mm were 4.9% and 3.6%, respectively. On the other hand, the simulation performed using a mesh with an element size of 0.15 mm was approximately 4.8 times longer than the simulation performed using a mesh with a 0.3 mm element size. When the element size is increased in simulations, there is a difference in obtained mechanical properties, although it is relatively minor compared to the significant decrease in computational time. Therefore, the element size of 0.3 mm was selected for further consideration, forming a mesh whose details are shown in Figure 8 on three different lattice configurations. In addition, the compressive strength estimated using an element size of 0.3 mm is 17% higher when compared to an element size of 0.15 mm. This difference in compressive strength implies higher sensitivity to mesh size, making estimation of compressive strength using this approach more challenging and less reliable.

**Figure 9:**
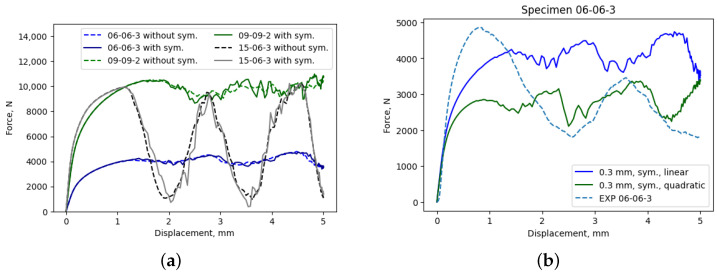
Comparison of load–displacement curves obtained (**a**) with and without symmetry boundary conditions and (**b**) with linear and quadratic elements.

The investigation into the potential reduction in computational costs during the simulation of the mechanical response of lattices has led to the consideration of one-quarter symmetry boundary conditions. The applicability of these boundary conditions, which significantly reduce computational costs, was assessed on three different lattice configurations. Based on the load–displacement curves depicted in Figure 9a, it is evident that there is an overlap in the initial part of the load–displacement curve. Once damage is initiated, at displacements exceeding 1 mm, slight differences between load–displacement curves with and without symmetry conditions can be observed. Since the symmetry condition resulted in a reduction in computational time from 5.50 h to 1.48 h for simulations performed using a 0.3 mm element size, the differences in load–displacement curves after damage were considered to be of minor relevance. Notably, since Young’s modulus and yield stress were determined from the initial segment of the curve, where damage has not yet occurred, the symmetry boundary condition will not significantly affect those results.

According to the simulation performed using a mesh with an element size of 0.3 mm with symmetry boundary conditions, Young’s modulus, yield strength, and compressive strength were 594.7 MPa, 5.7 MPa, and 10.6 MPa, respectively. When these results were compared with results of mesh with 0.3 mm element size, without symmetry boundary condition (Table 7), the differences in Young’s modulus, yield strength, and compressive strength were 1.2%, 0%, and 2.9%, respectively. This confirms that the symmetry boundary condition has a negligible impact on mechanical response when there is no significant plastic deformation or damaged elements. Therefore, the symmetry boundary condition was selected for further consideration to reduce computational time.

The sensitivity of Young’s modulus, yield strength, and compressive strength on element order have also been investigated. For this reason, one-quarter of the model with applied symmetry boundary conditions was meshed using quadratic tetrahedral elements (C3D10). These elements are generally less stiff than linear tetrahedral (C3D4) elements. The results obtained with quadratic elements show higher deviation from experimental data than those obtained with linear elements. Furthermore, the computational time needed to complete the simulation using quadratic elements is substantially longer (Table 7). Therefore, first-order elements with an element size of 0.3 mm were chosen for all simulations, along with a one-quarter symmetry boundary condition. While beam elements can further reduce computational costs, they present additional challenges in accurately modeling contact between neighboring struts, capturing local effects, and incorporating damage initiation and evolution laws. These factors complicate the accurate simulation of crushing behavior during the collapse of adjacent unit cells and layers. Despite these challenges, the beam elements can provide satisfactory results, particularly in cases where slender struts are used in unit cell geometries [25].

As shown in Figure 9b, in the linear-elastic region, there is reasonable agreement between force-displacement curves, while, in the plastic region, there is a significant difference. Interestingly, this observation aligns with findings reported by Lozanovski et al., who came to the same conclusion regarding element order and size selection [17].

In our research, Young’s modulus obtained from the model utilizing quadratic elements was found to be −8.9% lower compared to the calculation based on linear elements. Furthermore, the yield strength derived from the model utilizing quadratic elements was lower by −10.5% compared to the yield stress calculated using linear elements.

## 5. Results and Discussion

**Figure 10:**
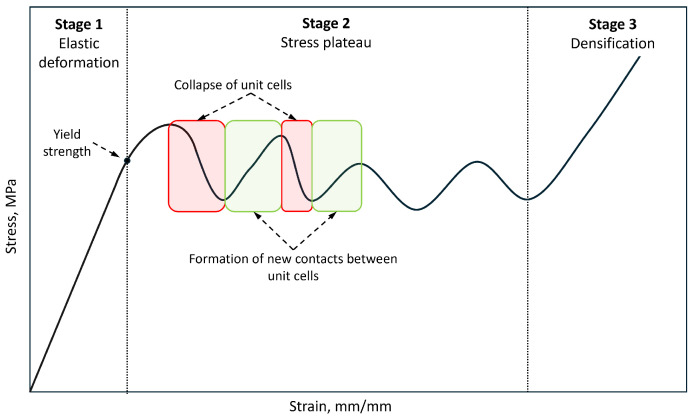
Typical compressive behavior of BCC lattice structures.

Typically, the compressive behavior of the lattice structures composed of BCC unit cells has three stages [1], as illustrated in Figure 10. The first stage is characterized by elastic deformation up to the yield strength. The second stage follows, characterized by a stress plateau during which stress fluctuations of varying intensity occur. These fluctuations result from the cyclic collapse of the unit cells and the formation of new contacts between neighboring cells. The intensity of these fluctuations and the width of the stress plateau can vary depending on the number of unit cell layers and their geometry. Additional loading leads to the third stage, characterized by increased stress due to the consolidation of the damaged unit cells. Those three stages have also been identified in the BCC and FCC lattice structures manufactured from the Ti6Al4V alloy [4].

**Figure 11:**
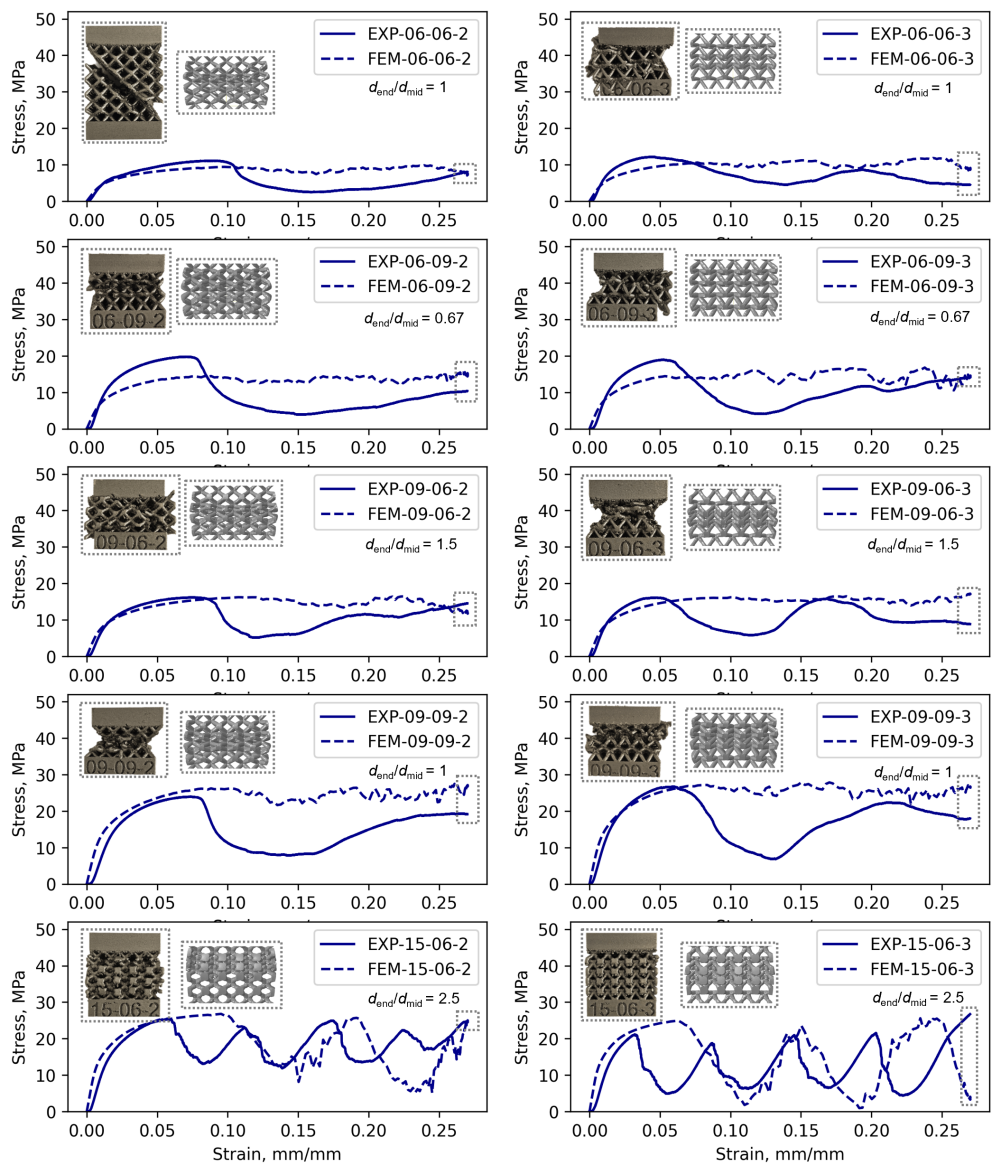
Comparison of stress–strain curves and collapse at the end of the test: experimental vs. numerical results.

The stress fluctuations were particularly pronounced in specimen configurations 15-06-2 and 15-06-3, as shown in Figure 11. This behavior is particularly pronounced in these configurations as the unit cells sustain damage within the layers, leading to layer collapse upon further loading. This leads to stress fluctuations. In the case of the experimentally determined mechanical response for both configurations, it is evident that there are four peaks indicating the collapse of each layer of unit cells, which can also be observed in the inset images of the lattice within the gray rectangle. For the simulated mechanical responses, the collapse of two layers was observed for the 15-06-2 configuration and three layers for the 15-06-3 configuration, which is also visible in the inset image within the gray rectangle showing the damaged lattice at the end of the simulation. Consequently, the proposed FE approach did not capture the collapse of each layer of unit cells, as observed in the experimental tests.

The remaining lattice structure configurations did not exhibit stress fluctuations from layer-wise collapse as the unit cell layers did not stack on top of each other upon failure. Instead, the failure occurred through different mechanisms. In these cases, the failed unit cells broke into smaller parts, with some fragments detaching from the specimen. For the 06-06-2 configuration, the damage progressed along an oblique plane, leading to the sliding of the unit cells along that plane. As a result, the mechanical response of these specimens differs from those observed in configurations 15-06-2 and 15-06-3.

BCC lattice structures are characterized by bending as the dominant deformation mechanism, resulting in lower ultimate compressive strength and Young’s modulus values compared to the FCC lattice, whose unit cells are subjected to stretching or compression [4]. In general, BCC lattice structures have substantial qualitative advantages for energy absorption applications [26], making them suitable for use in components designed for impact energy absorption [27]. Furthermore, Jin et al. reported that BCC lattice structures created from Ti6Al4V alloy using the LB-PBF process are more sensitive to heat treatment temperatures compared to FCC structures [4]. As a result, the deformation modes and the corresponding energy absorption capabilities of BCC structures can be further influenced by heat treatment, in addition to changes in their geometry.

As shown in Figure 11, the most pronounced stress fluctuations occur when the highest $d_{\mathrm{end}}/d_{\mathrm{mid}}$ ratio of 1.5/0.6=2.5 is used. In this case, the stress fluctuations are evident in both the symmetric (h=2 mm) and asymmetric (h=3 mm) unit cells. On the other hand, when the diameter at the strut midpoints is equal to or larger than the diameter at the strut endpoints $\left(d_{\mathrm{end}}/d_{\mathrm{mid}}\leq 1\right)$, the stress fluctuations are less significant. This is consistent with the findings of Liu et al. [28], who demonstrated that the stress fluctuations in BCC lattice structures during progressive damage can be controlled by managing shear band formation. Their research showed that adjusting the strut geometry, specifically by modifying the strut diameter ratios, effectively reduces shear band development. This is achieved by increasing the diameters at the strut midpoints and decreasing them near the nodes [28]. Such optimization significantly improves the energy absorption capacity, which is an important factor for energy-absorbing components [28]. When comparing configurations 15-06-2 and 15-06-3, the lattice structure with the asymmetric unit cell (configuration 15-06-3) exhibits noticeably higher stress fluctuations. Therefore, these configurations are not recommended for energy absorption applications as they lack the stable stress plateaus necessary for efficient performance in energy-absorbing components [28].

As depicted in Figure 11, a notable difference exists between the stress–strain curves obtained experimentally and numerically. Moreover, the post-damage stress fluctuations were not reliably captured using the proposed FE approach. For instance, in specimen 15-06-3, both the numerical and experimental data show cyclic behavior, yet the onset of cyclic behavior in the numerical simulations occurs later compared to the experimental observations. Several factors may contribute to the differences in the experimentally determined and numerically estimated mechanical properties.

Firstly, variations in the struts’ diameters, as well as the presence of defects, could be a key factor. After manufacturing, the actual diameters of the struts often differ from those specified in the CAD model. The dimensional accuracy of lattice structures similar to those tested in this study was analyzed in [20]. In that study, the identical apparatus was employed for specimen manufacturing using similar process parameters, and the AlSi10Mg powder used exhibited a similar particle size distribution. Lattices with 4 mm and 2 mm cubic unit cells featuring an FBCCZ topology, 3D printed in AlSi10Mg and 316L alloys, were examined. The measurements revealed that the strut diameters were larger than expected. The dimensional error was moderate for the vertical struts, remaining within ±0.15 mm. However, it was greater for the inclined struts, showing a systematic error of about 0.1 to 0.2 mm. The inclined struts were particularly affected by the staircase effect and the undesired adhesion of partially unmelted powders, leading to a reduction in the cell porosity. These factors could cause discrepancies between the FE simulations and experimental results. To enhance the prediction accuracy, in this work, mean strut diameter adjustments were incorporated into the models used in the FE simulations, as reported in Table 5. This approach does not consider the variations in the cross-sectional area across the different regions of the strut, nor the irregularities in shape that may arise due to the LB-PBF process. As reported in Table 8, the representative diameters used in the FEA for the 06-06-3 lattice configuration significantly affect the mechanical properties. The mean diameter ($\bar{d}$) and standard deviation ($\sigma_{d}$) were calculated from 12 measurements using a caliper. Subsequently, the impact of the strut diameter variations on the numerically estimated mechanical properties was evaluated using three characteristic diameters: $\bar{d}-\sigma_{d}$, $\bar{d}$, and $\bar{d}+\sigma_{d}$.

Evidently, varying the strut diameter between 0.71 mm and 0.83 mm results in a wide range of mechanical properties, with *E* varying from 147.9 MPa to 681.3 MPa, $R_{p0.2}$ from 1.9 MPa to 6.3 MPa, and $R_{m}$ from 3.6 MPa to 11.4 MPa. Notably, the FEA results for the mean diameter (0.77 mm) underestimate the experimental results for the same diameter. These results indicate that even minor errors in diameter measurements have a substantial effect on the values of the estimated mechanical properties. Furthermore, geometrical irregularities in actual struts act as stress concentrators and potential regions for damage initiation, ultimately reducing the mechanical performance of the lattice structure. To address this concern, struts can be subjected to scanning using μ-CT techniques and then integrated into the CAD model, following the method outlined in [25]. In this way, the actual strut features after manufacturing can be included in the model, enhancing its accuracy. However, this approach introduces additional financial, time, and computational costs. μ-CT techniques generate high-resolution models that capture manufacturing defects in high detail [17]. Incorporating all these defects into FE simulations requires an extremely fine mesh, which significantly increases the computational time [17]. To balance accuracy and computational efficiency, Lozanovski et al. proposed an AM-representative geometry that mimics the actual struts by preserving the key features in a smoother form, providing a compromise between precision and computational cost while performing FEA [17]. Nonetheless, the trade-offs in cost, time, and computational resources necessitate careful consideration to balance accuracy with practical feasibility in the design processes. In addition, the LB-PBF process creates fillets at the strut joints, influencing the mechanical response. Strategies to address this include increasing the strut diameter at the joints if beam elements are used or incorporating spheres when solid elements are used [17,29]. Moreover, defects in those parts produced using the LB-PBF process significantly affect their final properties. As demonstrated by Galy et al. [30], the primary defects observed during the LB-PBF of the AlSi10Mg alloy include porosity, surface roughness, anisotropy, and hot cracking. These defects can influence the experimental campaigns performed on LB-PBF parts. For example, pore size is a key indicator of material integrity, with larger “macroporosities” being more detrimental than smaller microporosities as they make the material more fragile. Poorly optimized process parameters can also result in high surface roughness, which reduces fatigue life [31]. Hot cracking is another critical issue, which can be mitigated by altering the alloy composition or optimizing the process parameters. Additionally, due to the complex thermal history of the LB-PBF process, parts often exhibit anisotropic mechanical properties, meaning their strength and performance vary depending on the build direction.

Secondly, the orientation of the struts during manufacturing may introduce additional discrepancies between the experimentally determined and numerically estimated mechanical properties as different build orientations affect the material’s ductility [32]. In this study, the mechanical properties of the parent material were assumed to be the same for all the struts regardless of their actual orientation based on the experiments from Li et al. [21], who performed tensile tests on vertically oriented struts with a diameter of 1 mm. By using these properties in the FEA, variations in ductility due to different orientations were not considered, contributing to the observed differences between the experimental and numerical results. In addition, Limbasiya et al. [33] demonstrated that the process parameters significantly influence the material density and mechanical properties. The mechanical properties employed in the FEA from [21] were determined on specimens manufactured using process parameters that differ from those utilized in this study. This disparity may further contribute to the observed discrepancies between the numerically estimated and experimentally determined mechanical properties.

Thirdly, disparities between numerical and experimental results arise from the selection of the element order and size, aiming to balance accuracy and computational cost. As shown in Table 9, the selection of the element order (linear vs. quadratic) has a pronounced effect on the estimated mechanical properties. The *E* obtained from the quadratic elements was reduced by −8.9% compared to that from the linear elements. Similarly, the $R_{p0.2}$ calculated using the quadratic elements was lower by −10.5% relative to the value derived from the linear elements. Furthermore, the $R_{m}$ showed an even greater reduction of −33% when quadratic elements were employed.

Therefore, as the element order increases, decreases in *E*, $R_{p0.2}$, and $R_{m}$ are observed, leading to a more pronounced discrepancy between the experimental and numerical results. This trend aligns with the findings of Lozanovski et al. [17]. In general, quadratic elements are less stiff than linear elements and tend to produce lower stress values.

The experimentally determined and numerically estimated mechanical properties are reported in Table 10. Interestingly, when specimen configurations with smaller diameter combinations were considered, the estimated Young’s modulus values using the proposed FE method were lower than the experimentally determined ones. However, for the larger-diameter combinations, the Young’s modulus values estimated using the proposed FE method were higher than the experimental ones. This is also true when the results of ultimate compressive strength are compared. Furthermore, the yield strength estimated using the proposed FE method in each case is lower than the experimentally determined one. In the initial design phase, when analyzing the potential configurations of lattice structures, this estimation, where the yield strength estimated using the FE approach is consistently lower than the experimentally determined value, prioritizes safety considerations.

Deviations between the numerically and experimentally observed mechanical responses of the BCC and graded-strut body-centered cubic (GBCC) unit cells were observed as well by Bai et al. in their work [6]. They identified two primary reasons for these discrepancies. First, the partially melted powder particles adhere to the surface, resulting in an increased strut diameter. Second, residual stresses develop in components produced using LB-PBF technology. Furthermore, Lozanovski et al. compared the mechanical properties of the two unit cell types, utilizing both idealized and AM-representative geometries through the FEA, with the experimental results [17]. For lattices composed of 4×4×6 unit cells with a face-centered cubic geometry with Z-struts, they reported a 90% difference in the Young’s modulus of the idealized lattice compared with the experimental results. Furthermore, in the case of the AM-representative geometry, they reported a 56% difference in Young’s modulus and a 33% difference in yield strength. In the case of the FCC lattices, they reported large errors as well. In our study, the most significant difference between the experimentally and numerically determined Young’s modulus values occurred in the 15-06-3 configuration, with a discrepancy of 66.3%. Concerning the yield strength, the largest difference of −39.9% occurred in the case of the 06-09-3 configuration. Despite the efforts presented in this work, as well as those by Lozanovski et al. [17] and Bai et al. [6] on various lattice structures, a significant discrepancy between the numerically estimated and experimentally determined mechanical responses remains, underscoring the need for further investigation.

## 6. Conclusions

The proposed FE approach enables modeling the elastic and plastic behaviors of lattice structures under compressive loading conditions up to the ultimate compressive strength. The elastic modulus, a fundamental property of lattice structures, showed the lowest dependency on the order of the finite elements. However, the accuracy of its estimation relies on the approach used to reconstruct the actual strut size, geometry, defects, and on extracting the actual mechanical properties of the material. Therefore, discrepancies between the experimentally determined and numerically estimated mechanical properties highlight the need for refined modeling techniques and considering manufacturing variations. However, the proposed FE approach can be used to select the appropriate type of unit cell in the early stages of product development. In that case, the proper order and size of the finite elements should be selected as they have a significant influence on the estimated yield strength, ultimate compressive strength, and mechanical response of the lattice structure during strut damage progression. Moreover, a substantial decrease in computational time while maintaining accuracy can be achieved by applying the one-quarter symmetry boundary condition.

Significant variations in Young’s modulus, yield strength, and ultimate compressive strength were observed across the different specimen configurations, as confirmed by the experimental and numerical analyses. Furthermore, the lattice structures with symmetric unit cells reached their ultimate compressive strength at higher strain than those with asymmetric unit cells. This observation implies that lattice structures with symmetric unit cells might be advantageous in applications requiring a higher deformation capacity before the initial failure occurs. The proposed FE approach estimated lower Young’s modulus values for the configurations with thinner struts than the experimental results. In contrast, the FEA provided a higher Young’s modulus for the configurations with thicker struts than what was determined experimentally. The FE method also consistently underestimated the yield strength compared to the experimental outcomes, with some configurations showing particularly large discrepancies.

While the findings provide valuable insights, it is acknowledged that future work will prioritize expanding the sample size and incorporating additional repetitions to enhance the robustness and generalizability of the results. Furthermore, the reason for the quadratic elements exhibiting solutions that deviate more from the experimental data compared to the linear elements requires further investigation.

## Figures and Tables

**Table 1:** Unit cell configurations based on the fractional factorial design with three levels of $d_{\mathrm{end}}$ and one replicate per configuration.

Specimen ID	$d_{\mathrm{end}}$, mm	$d_{\mathrm{mid}}$, mm	*h*, mm
06-06-2	0.6	0.6	2
06-06-3	0.6	0.6	3
06-09-2	0.6	0.9	2
06-09-3	0.6	0.9	3
09-06-2	0.9	0.6	2
09-06-3	0.9	0.6	3
09-09-2	0.9	0.9	2
09-09-3	0.9	0.9	3
15-06-2	1.5	0.6	2
15-06-3	1.5	0.6	3

**Table 2:** Chemical composition of the AlSi10Mg powder.

Chemical Element	Al	Si	Mg	Fe	Ti	Zn	Other
Content, %wt	Rest	10.38	0.26	0.17	0.02	0.01	<0.03

**Table 3:** Process parameters employed for producing the samples.

	Surface	Contour I	Contour II
Laser power	200 W	200 W	200 W
Scanning speed	800 mm/s	1250 mm/s	350 mm/s
Spot diameter	140 µm	100 μm	50 μm
Layer thickness	25 µm	25 μm	25 μm
Hatch distance	112 µm	-	-

**Table 4:** Mechanical properties determined experimentally.

Specimen ID	$E_{\exp}$, MPa	$R_{p0.2,\exp}$, MPa	$R_{m,\exp}$, MPa
06-06-2	552.5	5.9	11.1
06-06-3	824.7	8.9	12.2
06-09-2	1120.9	13.1	19.8
06-09-3	1118.4	13.8	18.9
09-06-2	908.6	10.4	16.1
09-06-3	1038.7	12.6	16.1
09-09-2	1113.1	17.1	23.9
09-09-3	1354.7	21.1	26.7
15-06-2	1364.7	15.6	25.4
15-06-3	1349.1	16.1	21.2

**Table 5:** Designed and actual strut diameters after PBF-LB.

Designed Diameter, mm	Actual Mean Value, mm	St. Dev., mm	CoV, %
0.6	0.77	0.06	8.4
0.9	1.05	0.13	12.2
1.5	1.62	0.04	2.8

**Table 6:** Mechanical properties for different element sizes.

Element Size, mm	*E*, MPa	$R_{p0.2}$ MPa	$R_{m}$ MPa	Computational Time, h
0.15	560.3	5.5	8.8	26.55
0.2	581.4	5.7	9.6	13.26
0.3	594.7	5.7	10.6	5.50
0.6	606.1	5.9	10.3	5.75
1	608.3	5.7	10.6	3.49

**Table 7:** Mechanical properties for linear and quadratic tetrahedral elements of the model with one-quarter symmetry.

Element Order	Element Size, mm	*E*, MPa	$R_{p0.2}$, MPa	$R_{m}$, MPa	Comp. Time, h
Linear—no sym.	0.3	587.7	5.7	10.3	5.50
Linear—sym.	0.3	594.7	5.7	10.6	1.48
Dif.	-	1.2%	0%	2.9%	−73.1%
Linear—sym.	0.3	594.7	5.7	10.6	1.48
Quadratic—sym.	0.3	541.8	5.1	7.1	17.18
Dif.	-	−8.9%	−10.5%	−33%	1060.8%

**Table 8:** Effect of diameter variations $\bar{d}-\sigma_{d}$, $\bar{d}$, and $\bar{d}+\sigma_{d}$ on numerically estimated mechanical properties for specimen configuration 06-06-3.

	FEA			Experiment
	$\bar{d}-\sigma_{d}=0.71$ mm	$\bar{d}=0.77$ mm	$\bar{d}+\sigma_{d}=0.83$ mm	$d_{\mathrm{actual}}=0.77$ mm
*E*, MPa	147.9	594.7	681.3	824.7
$R_{p0.2}$, MPa	1.9	5.7	6.3	8.9
$R_{m}$, MPa	3.6	10.6	11.4	12.2

**Table 9:** Effect of element order on numerically estimated mechanical properties for specimen configuration 06-06-3.

Element Order	*E*, MPa	$R_{p0.2}$ MPa	$R_{m}$ MPa
Linear	594.7	5.7	10.6
Quadratic	541.8	5.1	7.1
Difference	−8.9%	−10.5%	−33%
Experiment	824.7	8.9	12.2

**Table 10:** Comparison of mechanical properties obtained experimentally and numerically.

ID	$E_{\exp}$, MPa	$E_{\mathrm{fem}}$, MPa	Dif., %	$R_{p0.2,\exp}$, MPa	$R_{p0.2,\mathrm{fem}}$, MPa	Dif., %	$R_{m,\exp}$, MPa	$R_{m,\mathrm{fem}}$, MPa	Dif., %
06-06-2	552.5	493.3	−10.7	5.9	5.3	−10.2	11.1	9.2	−17.1
06-06-3	824.7	594.7	−27.9	8.9	5.7	−36	12.2	10.6	−13.1
06-09-2	1120.9	840.4	−25	13.1	8.0	−38.9	19.8	14.4	−27.3
06-09-3	1118.4	918.8	−17.8	13.8	8.3	−39.9	18.9	14.4	−23.8
09-06-2	908.6	910.8	0.2	10.4	9.2	−11.5	16.1	15.7	−2.5
09-06-3	1038.7	982.6	−5.4	12.6	9.3	−26.2	16.1	16.0	−0.6
09-09-2	1113.1	1601.0	43.8	17.1	15.2	−11.1	23.9	26.1	9.2
09-09-3	1354.7	1772.0	30.8	21.1	15.8	−25.1	26.7	27.2	1.9
15-06-2	1364.7	2079.8	52.4	15.6	15.4	−1.3	25.4	26.5	4.3
15-06-3	1349.1	2243.1	66.3	16.1	15.3	−5	21.2	24.9	17.5

## Data Availability

The original contributions presented in the study are included in the article, further inquiries can be directed to the corresponding author.
